# Efficacy of affirmative cognitive behavioural group therapy for sexual and gender minority adolescents and young adults in community settings in Ontario, Canada

**DOI:** 10.1186/s40359-021-00595-6

**Published:** 2021-06-07

**Authors:** Shelley L. Craig, Andrew D. Eaton, Vivian W. Y. Leung, Gio Iacono, Nelson Pang, Frank Dillon, Ashley Austin, Rachael Pascoe, Cheryl Dobinson

**Affiliations:** 1 Factor-Inwentash Faculty of Social Work, University of Toronto, 246 Bloor St. W., Toronto, ON M5S1V4 Canada; 2 Faculty of Social Work, University of Regina – Saskatoon Campus, Saskatoon, SK Canada; 3 School of Social Work, University of Connecticut, Hartford, CT USA; 4 College of Integrative Sciences and Arts, Arizona State University, Tempe, AZ USA; 5 Ellen Whiteside-McDonnell School of Social Work, Barry University, Miami, FL USA; 6 Planned Parenthood Toronto, Toronto, ON USA

**Keywords:** Sexual and gender minorities, Depression, Coping, Stress appraisal, Cognitive behavioural therapy, Community intervention

## Abstract

**Objective:**

This study tested the efficacy of AFFIRM, a brief affirmative cognitive-behavioural group intervention tailored to reduce psychosocial distress and improve coping among sexual and gender minority adolescents and young adults (SGMY).

**Method:**

SGMY (*n* = 138; *M* age = 22.44) were allocated to immediate 8-week AFFIRM intervention delivered at 12 community-based organisations or an 8-week waitlisted control. At baseline, post-intervention or post-waitlist, participants completed self-reported assessments of depression, hope, coping, and stress appraisal. Implementation outcomes of feasibility and acceptability were also assessed.

**Results:**

Compared to waitlist, SGMY in the intervention condition significantly reduced their depressive symptoms (*b* = − 5.79, *p* = .001) as well as increased reports of hope (agency: *b* = 0.84, *p* = .001; pathway: *b* = 0.79, *p* = .001), and coping by emotional support (*b* = 0.59, *p* < .001), instrumental support (*b* = 0.67, *p* < .001), positive framing (*b* = 0.59, *p* < .001), humour (*b* = 0.36, *p* = .014), planning (*b* = 0.49, *p* < .001) as well as reflective coping (*b* = 0.27, *p* = .009). Intervention participants were also less likely to perceive stress as a threat (*b* = − 0.43, *p* = .001), and more likely to perceive stress as challenge (*b* = 0.67, *p* < .001) and have the resources to deal with that stress (*b* = 0.38, *p* = .016) in comparison to waitlisted control participants. All outcomes had medium to large effect sizes. AFFIRM participants reported low attrition (8.5%) and high levels of engagement and acceptability (e.g. 99% agreed intervention was relevant to their lives). Over 63% of the community organizations that participated in the training hosted AFFIRM at least once during the study.

**Conclusions:**

Results demonstrate efficacy for the community-based implementation of an affirmative clinical intervention designed for SGMY to address depression and foster coping with universal and minority stressors.

**Supplementary Information:**

The online version contains supplementary material available at 10.1186/s40359-021-00595-6.

## Background

Sexual and gender minority adolescents and young adults, hereafter referred to as sexual and gender minority youth (SGMY) experience significant mental health disparities and psychological distress compared to their cisgender, heterosexual counterparts [1]. Extant research has identified troubling rates of depression and suicidality in this population [2]. The CDC Youth Risk Behaviour Survey found that over 60 percent of sexual minority youth did not engage in their typical activities due to sadness and hopelessness and were four times as likely to attempt suicide compared to heterosexual youth [3]. According to a recent metanalysis, sexual minority youth have three times the odds of developing a depressive disorder or symptoms compared to their peers [4]. In studies with transgender or gender diverse populations, over 40% attempt suicide during adolescence or young adulthood [5] and 49–62% of youth meet criteria for a depressive disorder [6]. Similarly, a national investigation found that sexual and gender minority college students (N = 72,815) were twice as likely to report depression that significantly impacts functioning and three times more likely to have suicidal ideation compared to their heterosexual and non-transgender peers [7].

The mental health disparities of SGMY are typically attributed to minority stress, described as the unique stressors emerging from a stigmatised sexual orientation [8] or gender identity [9]. Minority stress theory describes how discrimination contributes to internalised stigma (i.e., negative beliefs about one’s own sexual and/or gender minority status) [10], ultimately resulting in depression and mental health problems for SGM populations [2, 11]. SGMY are more likely to endure distinct minority stressors that exacerbate psychosocial distress such as familial rejection, victimisation, and social exclusion compared to their cisgender, heterosexual peers [1]. Minority stressors can contribute to feelings of hopelessness, which may partially explain elevated suicidality in SGMY [2, 12–14]. Contributing to the complexity of their psychological concerns, SGMY also experience higher rates of adverse childhood events than their non-SGMY counterparts [15]. Of note are the particularly high rates of childhood emotional abuse and neglect [16], which are linked to increased risk of suicidality and substance dependence in adolescence and adulthood [1]. Taken together, the experiences of minority stress and trauma can compromise the mental health and coping capacity of SGMY.

Stressors experienced by SGMY collectively impact cognitive, affective and behavioural processes [17, 18]. Stress is present when an event is perceived to exceed coping resources [19]. For SGMY, stress includes universal adolescent stressors as well as chronic and acute experiences of minority stress that can contribute to negative emotions, difficulty coping, and mental health problems [20]. The perception or cognitive appraisal of stress supports emotional regulation [21] and impacts long-term behavioural health [22]. Cognitive appraisals can take two primary forms: perceiving stress as a challenge or as a threat. Appraising stress as a challenge allows for positive reinterpretation that can contribute to personal growth, active coping, and healthy adjustment [23]. Appraising stress as a threat suggests the potential for harm has been linked to maladaptive coping, posttraumatic stress symptoms and depression [24]. Although appraisals and coping strategies utilised by SGMY have only recently come under investigation, they have particular relevance for interventions addressing mental health [25, 26]. As SGMY experience persistent minority stressors, attending to cognitive appraisals associated with experiences of discrimination may have a notable impact on overall psychosocial functioning [27, 28]. Affirmative interventions that mobilise SGMY coping skills to help them identify, evaluate, and interrupt the influence of minority stress on their behavioural health are increasingly influential [20, 25], yet research with large, community-based samples is needed.

Coping—described as conscious actions to regulate behaviour and thoughts when under stress [29]—has been identified as a mechanism to improve mental and behavioural health among SGMY [30, 31]. Several types of coping may have particular salience for SGMY. Emotional support coping revolves around engaging in self-care strategies (such as privately dressing in clothes that match one’s gender identity, even if unable to do so publicly) to prevent and mitigate feelings of anguish that can arise from stressful situations [32], such as incidents of minority stress. Instrumental support coping refers to leveraging personal relationships (such as speaking with another SGMY or SGM adult) to access support related to dealing with a challenging situation or environment [33]. Positive framing is the internal coping process of finding a positive/optimistic perspective (such as an opportunity for growth) in a problematic situation [34]. This type of coping aligns with the importance of meaning making in the face of structural stigma, a potentially important form of resilience for SGMY [35, 36]. Planning coping involves identifying a specific strategy (such as roleplaying ‘coming out’ to parents with a friend in advance) to manage potentially challenging situations in the future [33].

Hope, as a motivational and cognitive construct, has been described as consisting of two main elements, pathway and agency, that move individuals toward attaining their goals [37, 38]. Based on Snyder’s hope theory [38], pathway thinking refers to one’s perceived ability to create a roadmap toward achieving goals and problem solve obstacles along the way. Agency thinking refers to the willpower and determination to implement and sustain momentum towards desired goals. Hope has been associated with psychological well-being, life satisfaction, academic achievement, and lower levels of depression among youth [39–41]. Although hope has not been extensively studied in racially and ethnically diverse youth populations, hope has been associated with positive affect, life satisfaction, and support from loved ones among Mexican–American youth (n = 135) [37] while grounded theory research has identified importance of hope among transgender and gender diverse youth [42]. Hope and hopelessness are not exact inverses; rather, low hope and high hopelessness have been found to be distinct but correlated constructs [43]. Given that SGM populations report greater levels of hopelessness [28], improving hope may be an important target for intervention with SGMY as hope can be a resilience factor that protects against the negative impacts of hopelessness, such as suicidality [43].

## Affirmative cognitive behavioural therapy

Building on the efficacy of cognitive behavioural therapy (CBT) to treat adolescent mental health problems [44, 45], affirmative CBT, which actively validates stigmatised identities by acknowledging the impact of interpersonal and structural sources of SGM identity-based stigma and targets cognitive, affective and behavioural processes, is a promising approach to combatting the increased rates of psychosocial distress in young gay and bisexual men [20] and SGMY ages 15–18 [25]. For example, the Effective Skills to Empower Effective Men (ESTEEM) CBT curriculum significantly reduced depressive symptomology (*d* = 0.55) through its impact on universal factors such as social support and rumination in a sample of gay and bisexual men aged 18–35 [20]. Through a range of SGM-specific intervention skills, affirmative CBT approaches can effectively address complex stressors that exacerbate depression and psychological distress for SGMY by helping them evaluate the impact of stress on their wellbeing as well as mitigate feelings of self-blame and shame associated with stigma [4].

Emerging from extensive clinical work, an affirmative CBT group intervention (AFFIRM) was conceptualised to address the lack of evidence-informed programs tailored to the specific mental health needs of SGMY [46]. Although models such as ESTEEM that were developed for individual therapy with gay and bisexual men are important and efficacious [20], it is also critical to deliver group-based affirmative CBT that can address the needs of a range of community-based SGMY. Through a manualised curriculum, AFFIRM emphasises the principles of CBT while addressing the specific developmental needs of SGMY contending with a daily onslaught of minority stress [25]. AFFIRM provides opportunities for SGMY to be more aware of, address, and change cognition (self-awareness, identifying risk), mood (recognising the link between thoughts and feelings), and behaviour (identifying strengths and ways of coping). Through AFFIRM, SGMY learn to better understand sources of their minority stress (e.g., media messages, family rejection, school bullying), and engage in a variety of cognitive and behavioural strategies to unlearn, challenge, and question stigmatising messages and beliefs. This process is hypothesised to: (a) validate the authenticity of their challenging experiences; (b) improve youths’ ability to locate the problem outside of themselves (e.g., there is something wrong with homophobia/transphobia) rather than within themselves (e.g., there is something wrong with me); and (c) foster the development of healthy coping strategies (e.g., build affirmative support, challenge negative messages, engage in goal setting) that positively impact mental health.

AFFIRM was developed systematically in concert with SGM community members, using the ‘adapt and evaluate framework’ described elsewhere [47] and intended for delivery across a range of community contexts. Interventions developed from the ‘ground up’ are more likely to meet unique population needs, increase access to empirically validated care, be seamlessly adopted by agencies, and integrated into their existing service delivery contexts [25, 48, 49]. Given that most community-based agencies serve a wide range of youth identities (e.g., lesbian, gay, bisexual, transgender, and queer) and SGMY are more likely to seek support from community-based services than traditional mental health services [47], the AFFIRM study was designed to explore the intervention’s efficacy within ‘real-world’ service delivery settings. This approach is particularly innovative as community agencies for SGMY do not always have the capacity to deliver evidence-based mental health interventions and youth-serving organizations often do not have the knowledge or expertise to deliver tailored care to SGMY [46, 47].

## Present study

AFFIRM has shown positive outcomes in an open pilot feasibility study with: (a) large effects on depression (η^2^ = 0.22), reflective coping (η^2^ = 0.21), and threat appraisal (η^2^ = 0.18); (b) moderate effect on challenge appraisal (η^2^ = 0.15); and (c) small-to-moderate effect on resource appraisal (η^2^ = 0.04) from pre-intervention to three-month follow-up although eta-squared may overestimate effect sizes [25]. However, AFFIRM has not yet been implemented and evaluated on a sufficiently powered scale across a broader age range with a more reliable measure of effect size. To date, there has been a dearth of research exploring the efficacy of affirmative CBT interventions in community-based settings [50]. Given the urgent need for supportive interventions for SGMY [36], this study explored the efficacy of AFFIRM to reduce psychosocial distress with this population. Further, this study explored the impact of an affirmative CBT intervention on factors such as hope [42], coping [30] and stress appraisal [35] that may be particularly relevant to SGMY resilience and mental health [36] but have received less attention in intervention research with SGM populations. Specifically, AFFIRM participants are expected to report reduced depression and improved coping, stress appraisal and hope. This study is designed to fill practice and research gaps by simultaneously implementing AFFIRM in practice settings while evaluating its efficacy at reducing depression.

## Method

### Participants

From April 1, 2017 to February 1, 2020, participants were recruited through social media advertisements and flyers were circulated throughout professional network listservs (e.g., clinical psychologists and social workers) and local agencies (e.g., Planned Parenthood, SGM community centre). In addition, the organisations hosting AFFIRM (community health centres, youth centres, AIDS service organisations, and hospitals) distributed flyers to their staff and clients. SGMY were eligible to participate if they: (a) were 14–29 years old at time of enrolment; (b) self-identified as a sexual and/or gender minority; (c) were proficient in English. SGMY often endorse numerous options within sexual orientation (such as bisexual and pansexual) and gender identity (such as trans man and non-binary) categories given the fluidity of these identities in the context of their lives [51, 52]. For this study, non-mutually exclusive response options were provided: gender identity (cis man, cis woman, transgender (trans) man, trans woman, non-binary, agender, queer, and two-spirit), sexual orientation (gay, lesbian, bisexual, pansexual, queer, asexual/aromantic, straight, and not sure/questioning), ethnic/racial identity (White, Indigenous, Asian, Black, Hispanic/Latinx, Middle Eastern, and Multi-ethnic). To enable further analysis, this study added follow-up, forced response questions on most dominant sexual orientation and gender identity (same identity options as above) to determine if participants considered one of these identities to be more important than others. The most dominant identity was utilised to allow for comparisons in group membership and self-report assessments between conditions. Potential participants were excluded from the study if they were assessed to be in crisis (i.e., high risk of suicidality) or otherwise warranting a more intensive intervention. The study coordinator replied to screening survey participants to confirm eligibility and allocate individuals to the intervention or waitlisted control. All participants were able to continue to receive services from community agencies, which typically consisted of general support programs.

**Fig. 1:**
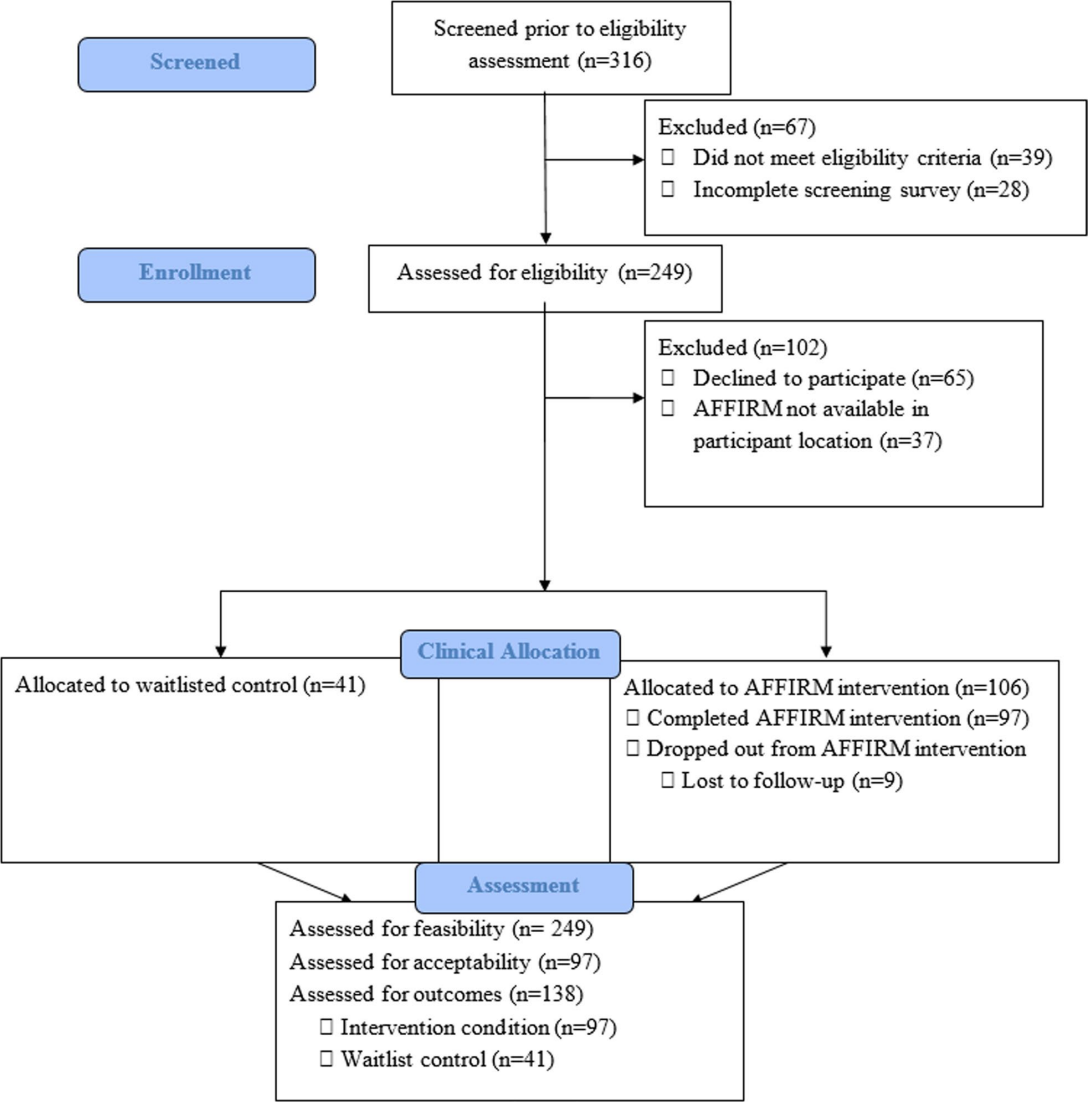
Participant flowchart

Figure 1 displays participant flow throughout the study. Out of 316 screened individuals, 249 (78.8%) were eligible for the study. From there, 65 declined to participate and 37 were in areas (mostly rural) where there were insufficient participants to hold a group. Therefore, a sample of 147 SGMY were allocated to the AFFIRM intervention (*n* = 106) and the waitlisted control (*n* = 41). The allocation process is described in *Study Design* subsection below. There were 9 SGMY who dropped out of the intervention, resulting in 97 completers from the intervention arm and a final sample of 138 SGMY participants.

As displayed in Table 1, the final sample of participants (*n* = 138) represented a wide range of gender, sexual orientation, and ethnic/racial identities. Participants’ ages ranged from 14–29 with a mean age of 22.44 years. Demographic comparisons between the intervention and control conditions found no significant difference between the two conditions regarding gender identity (χ^2^ = 13.06, *p* = 0.220), sexual orientation (χ^2^ = 16.21, *p* = 0.094), and most of the ethnic/racial identities (χ^2^ = 0.04–0.59, *p* = 0.442–0.852). The waitlist control was older (*t* = 2.51, *p* = 0.010) and consisted of a larger proportion of multi-ethnic participants (χ^2^ = 6.84, *p* = 0.009).

**Table 1:** Baseline characteristics

	AFFIRM intervention n(%)/Mean (*SD*) (*n* = 97)	Waitlist control n(%)/Mean (SD) (*n* = 41)	Condition comparisons
**Age (mean)**	21.88 (5.03)	23.78 (3.33)	*t* = 2.61, p = *.*010
**Gender identity (most important)**			χ^2^ = 13.06, *ns*
Transgender	22 (22.7)	9 (22.0)	
Cis woman	19 (19.6)	5 (12.2)	
Non-Binary	18 (18.6)	15 (36.6)	
Cis man	18 (18.6)	3 (7.3)	
Queer	11 (11.3)	1 (2.4)	
Agender	5 (5.2)	3 (7.3)	
Two-Spirit	1 (1.0)	1 (2.4)	
Other	3 (3.1)	4 (9.8)	
**Sexual orientation (most important)**			χ^2^ = 16.21, *ns*
Gay	26 (26.8)	6 (14.6)	
Queer	19 (19.6)	13 (31.7)	
Pansexual	17 (17.5)	5 (12.2)	
Bisexual	12 (12.4)	5 (12.2)	
Lesbian	12 (12.4)	2 (4.9)	
Straight	3 (3.1)	0 (0)	
ACE umbrella*	2 (2.1)	3 (7.3)	
Demi/demiromantic	2 (2.1)	1 (2.4)	
Not sure/questioning	2 (2.1)	3 (7.3)	
Other	2 (2.1)	3 (7.3)	
Ethnic/racial identity**			
White	46 (47.4)	21 (51.2)	χ^2^ = 0.17, *ns*
Asian	20 (20.6)	11 (26.8)	χ^2^ = 0.59, *ns*
Black	18 (18.6)	6 (14.6)	χ^2^ = 0.34, *ns*
Hispanic/Latinx	6 (6.2)	2 (4.9)	χ^2^ = 0.10, *ns*
Multi-ethnic	5 (5.2)	8 (19.5)	χ^2^ = 6.84, *p* = .009
Middle Eastern	4 (4.1)	2 (4.9)	χ^2^ = 0.04, *ns*
Indigenous	4 (4.1)	2 (4.9)	χ^2^ = 0.04, *ns*

*ACE Umbrella represents spectrum of asexuality

**Categories were not exclusive

### Procedure

#### Online screening survey

Recruitment materials described the intervention as an affirmative CBT-based group designed to help SGMY reduce depression and improve coping. These materials (i.e., flyer, social media posts) directed potential participants to the project website (www.projectyouthaffirm.org) where they completed a screening survey hosted by Qualtrics. The screening survey asked participants for their chosen name, age, contact information, gender, sexual orientation, pronouns, ability to attend AFFIRM, and preferred site. Eligible participants were contacted via email by the research coordinator and provided another Qualtrics link to a survey containing informed consent, study details and baseline measures (see primary outcome measures below).

#### Study design

After participants’ study eligibility was confirmed, participants then completed the baseline assessment. Following this, the study coordinator allocated participants to: (a) AFFIRM intervention; or (b) waitlisted control. Participants were allocated to the AFFIRM intervention immediately if there was sufficient registration (minimum six) at a single site to commence a viable group that was comprised of developmentally appropriate group composition with session examples appropriately tailored to group membership. Participants in a single group varied in age by no more than five years, with cut-offs of 14–18 (4 groups), 19–24 (7 groups), and 25–29 (10 groups) to reflect differences in life stage. Group size ranged from 4 to 14 participants with a mean of 8.71, median of 9, and mode of 9. If recruitment for a developmentally appropriate group of sufficient size would take longer (such as in more rural areas), participants were allocated to the waitlisted control and offered local resources. This method of allocation was used as opposed to traditional randomisation which can be unethical for vulnerable populations, unacceptable to community sites, and not feasible in group intervention studies where groups recruit, enrol, and commence regularly over a sustained period of time [53, 54]. As AFFIRM has shown promise in pilot testing [25] and as SGMY are a vulnerable population, allocating participants solely to an inactive control group could unethically deny them of a helpful intervention [55, 56]. While participants knew the results of their allocation to the AFFIRM or waitlisted control conditions, care providers (i.e., facilitators) were masked to the content of participant surveys. Study procedures were approved by the University of Toronto’s Health Sciences Research Ethics Board (Protocol ID# 35,229) and the study is registered on clinicaltrials.gov at NCT04318769.

Participants assigned to the AFFIRM condition completed their first session immediately after baseline assessment, while those in waitlist control continued their usual care and received e-mails to remind them of their upcoming groups. Both the AFFIRM intervention and waitlist period lasted for two months. The AFFIRM condition completed post-intervention surveys on Qualtrics. Followed by a two-month wait, waitlisted control participants completed post-waitlist surveys. Baseline data (i.e., pre-intervention or pre-waitlist) was assigned as time = 0 and post-test data (i.e., post-intervention or post-waitlist) was assigned as time = 1. For process outcomes, descriptive statistics of feasibility and acceptability measures were obtained and reported.

#### Intervention

The AFFIRM intervention protocol has been previously described [25, 57] and consisted of a two-hour orientation followed by eight weekly, two-hour group sessions. The orientation session described group norms (such as confidentiality, respect for difference, etc.) and detailed the AFFIRM curriculum so that potential participants were fully informed. In summary, sessions 1–2 focus on an overview of CBT and the impact of minority stressors; session 3–4 describe key approaches to cognitive restructuring; sessions 5–6 review coping skills, behavioural activation and build skills to develop hope and set goals and sessions 7–8 engage participants in an assessment of their social support network, develop self-compassion and integrate their new skills into their future plans. Each of the eight group sessions addressed a particular topic (e.g., thought stopping, behavioural activation) and featured: (a) a group check-in and review of previous sessions and homework; (b) an overview of the current session’s objectives; (c) introduction of behavioural activities; (d) practice and rehearsal of behavioural activities; and (e) reflective check-out and session summary [25]. Minor modifications of the examples used were made to ensure the intervention was relevant to young adults (e.g., scenarios were adapted to college or work instead of high school settings, living alone or with partners instead of parents). Twelve sites that served youth or SGMY populations in urban areas of the Canadian province of Ontario (community health centres, youth centres, HIV/AIDS service organisations, and hospitals) were actively engaged to host (a minimum of one group) with a total of 21 groups delivered. Sites were a mix of service settings (e.g., Sherbourne Health Centre, Compass Community Health, Hamilton Family Health Team, the Black Coalition for HIV/AIDS Prevention (Black CAP), and the Alliance for South Asian AIDS Prevention (ASAAP) with just one LGBTQ + specific organization (the 519 Church Street Community Centre). AFFIRM was delivered by two trained facilitators that were intentionally paired. The first was employed by the principal investigator as a graduate-level clinician on the AFFIRM research team and the second was employed by the host site as part of the goal to support community capacity to deliver empirically based mental health care to SGMY. Facilitators predominantly identified as members of the SGM community with a range of sexual, gender, racial, and ethnic identities.

#### Treatment supervision and fidelity

The first and fourth authors, both licensed social workers, supervised AFFIRM’s delivery through weekly individual and group supervision meetings. Facilitators completed weekly process notes. All AFFIRM sessions were audio-recorded for supervision and fidelity analysis. Licensed clinicians trained in AFFIRM (but not facilitating) utilised a modified CBT fidelity checklist [58] to analyse session recordings, which were discussed with facilitators in supervision.

#### Numbers analysed

Primary outcomes (depression, reflective coping, stress appraisal, coping, and hope) were assessed within a sample of 138, by comparing pre- and post-intervention timepoints of the intervention condition (*n* = 97) with pre- and post-waitlist timepoints of the waitlisted control (*n* = 41). No significant difference was found among most of the baseline scores (pre-intervention timepoint for intervention condition; pre-waitlist timepoint for control condition) of our primary outcomes between the two groups (*t* = 0.29–1.80, *p* = 0.076–0.772), except emotional support coping (*t* = 2.34, *p* = 0.022), instrumental support coping (*t* = 2.87, *p* = 0.005), and substance use coping (*t* = 3.19, *p* = 0.002). Youth from the control condition had higher baseline scores in all three coping strategies than the intervention condition. Acceptability was assessed from the 97 completers who completed the post-intervention satisfaction scale.

#### Outcome measures

Five standardised measures, described below, were administered via online Qualtrics surveys at pre-waitlist (if applicable), pre-intervention, and post-intervention. Refer to Additional file 1 for the survey questionnaire.

*Beck depression inventory-II (BDI-II)* The BDI-II is a 21-item measure of depression in adolescents and adults [59] which has been utilised with SGMY [60] and validated in youth as young as 13 years old [61]. Scale items are self-reported based on the previous two weeks with total scores ranging from 0 to 63 (higher score indicates higher level of depression). Questions such as “I feel sad most of the time” are scored on a scale from 0 to 3. Higher total scores indicate more severe depression, with standardised values (0–13 minimal depression; 14–19 mild depression; 20–28 moderate depression; 29–63 severe depression). The BDI-II has high test–retest reliability (*r* = 0.93) and evidence of validity [59, 62]. In the present study, the scale demonstrated excellent internal consistency (baseline α = 0.92; post-test: α = 0.93).

*Proactive coping inventory for adolescents-A (PCI-A): reflective coping subscale (RCS)* The RCS is an 11-item subscale of the PCI-A which measures the process of coping at cognitive and behavioural levels in daily life [63]. Respondents select scores from 1 (*not at all true*) to 4 (*completely true*) in questions such as “I take action only after thinking carefully about a problem”. A higher score indicates more intention to practice reflective coping. Similar to previous research (α = 0.88) [63], the RCS demonstrated evidence of good reliability (baseline α = 0.88; post-test: α = 0.90) in the present study.

*Brief COPE inventory* The 28-item Brief COPE Inventory (BCI) is designed to measure how people deal with stress in their lives [64] and has been validated with 18–24-year-old SGMY [65], and adolescents starting at age 12 [66]. Scale items are endorsed from 1 (*not at all*) to 4 (*a lot*) and a sample item is “When stressed generally in my daily life, I get help and advice from other people.” There were 14 subscales in the BCI, and we excluded five subscales (self-distract, denial, venting, and acceptance, religion) because of unsatisfactory reliabilities (lower than 0.60). The 9 remaining subscales were used as primary outcome variables. A higher score in the subscale represents higher intention to cope using that specific strategy. Similar to Carver’s original work [64], the reliabilities for our study, noted in parenthesis, indicated good to marginally acceptable evidence of satisfactory reliability: active coping: α = 0.68 (baseline α = 0.74; post-test α = 0.70); substance use: α = 0.90 (baseline α = 0.97; post-test α = 0.94); emotional support: α = 0.71 (baseline α = 0.85; post-test α = 0.88); instrumental support: α = 0.64 (baseline α = 0.81; post-test α = 0.82), behavioural disengagement: α = 0.65 (baseline α = 0.75; post-test α = 0.69); positive framing: α = 0.64 (baseline α = 0.81; post-test α = 0.77); planning: α = 0.73 (baseline α = 0.70; post-test α = 0.72); humour: α = 0.73 (baseline α = 0.88; post-test α = 0.88) and self-blame: α = 0.69 (baseline α = 0.80; post-test α = 0.83).

*Stress appraisal measure for adolescents (SAMA)* The 13-item SAMA assesses daily stress appraisal as challenge, threat, and resources [22]. Responses range on a 5-point scale (1 = *strongly disagree* to 5 = *strongly agree*), and a sample item is “In my daily life, I have the ability to overcome stress”. Higher scores represent higher likelihood to appraise stress as challenge, rather than threat, and have the resources to meet the challenge. The measure has been adapted for Latinx adolescents [67] and has shown evidence of good internal reliability (Challenge: α = 0.79, Threat: α = 0.81; Resources: α = 0.79) [22]. In the present study, reliabilities of challenge, threat, and resources subscales were α = 0.80, α = 0.81 and α = 0.77 respectively for baseline and α = 0.79, α = 0.85 and α = 0.85 for post-test.

*Hope scale (HS)* The HS is a 12-item measure of dispositional hope [68] assessed on an 8-point scale from 1 = *definitely false* to 8 = *definitely true* with a higher score representing more hope; originally developed for adults, validation studies have found the HS to be unidimensional with youth and young adults starting at age 13 [69] and 14 [70]. The scale consists of two 4-item subscales measuring pathway (tendencies to plan ways to meet goals) and agency (goal-directed determination) [68]. A sample item is “My past experiences have prepared me well for my future.” The original reliabilities were α = 0.76 (agency subscale), and α = 0.80 (pathway subscale) with university students [68]. For this study, good internal consistencies were found: agency (baseline: α = 0.85; post-test: α = 0.85) and pathway (baseline: α = 0.82; control: α = 0.82).

#### Implementation measures

AFFIRM’s community implementation necessitated an assessment of feasibility and acceptability. Feasibility was measured by calculating two proportions. The first proportion was the number of sites approached to offer AFFIRM compared to how many sites implemented AFFIRM once, and how many continued to implement AFFIRM two or more times. The second proportion was the number of participants that enrolled, commenced, and completed each iteration of the intervention. Feasibility was assessed against a priori targets that the research team agreed upon based on feasibility targets of similar trials that utilised similar recruitment and intervention types [71, 72]. The a priori-based targets were that 75% of eligible participants would be interested in participating, 60% of those would complete the initial assessment, and 55% of those would complete the intervention and post-intervention assessment.

Acceptability was assessed by the AFFIRM Acceptability Survey, a 17-item, 4-point Likert-style questionnaire (1 = *strongly disagree* to 4 = *strongly agree*). Items address intervention utility, relevance, and overall satisfaction [25]. Open-ended questions that assessed the least and most helpful aspects of AFFIRM were included.

### Analytic plan

For primary outcomes, the missing data ranged from 0 to 2.2% for pre-waitlist/ pre-intervention measures and 0 to 4.3% for post-waitlist/post-intervention measures. The Little’s Missing Completely at Random (MCAR) Test indicated that the data were missing completely at random (*χ*^2^ = 2736.72, *p* = 1.00). Missing items of the BDI-II were imputed using the Expectation–Maximization (EM) Algorithm in SPSS 26. Average scores were calculated for all other measures, so if a participant missed some items within a scale, the average scores of the remaining items were calculated. For all scales, if the participant missed the whole scale, the missing data were handled by pairwise deletion [73]. For acceptability items, missing data ranged from 5.2 to 10.3% and was imputed using the EM Algorithm.

First, demographic variable and primary outcome baseline comparisons between the intervention and control conditions were analysed using independent samples t-tests (for continuous variables such as age and depression score) and chi-square tests (for categorical variables such as gender identity and ethnic/racial identity). Next, we conducted linear mixed models with restricted maximum likelihood estimation (REML) to test the effects of Time (baseline = 0, post-test = 1), Condition (control = 0, intervention = 1), and Time X Condition for all primary outcomes (depression, reflective coping, stress appraisal, coping, and hope). Age (centred at the mean of the sample) was included as a covariate variable in the model to account for the age difference between the two conditions. The two measured time points were nested under each individual; since there was possible non-independence among participants in terms of therapy group, participants were further nested under their AFFIRM group. Intercepts and slopes of the two conditions were estimated and tested for statistical significance. Due to multiple testing, the significance of coefficients were adjusted by controlling the false discovery rate (FDR) [74, 75]. Cohen’s *d*s were used to represent the effect sizes of the interaction terms, calculated using the *t* statistics of the interaction term (*d* = 2*t*/$\sqrt{df}$).

## Results

### Outcomes

Table 2 shows the descriptive statistics of baseline and post-test scores for both intervention and waitlisted control conditions. Results of the linear multilevel models are presented in Table 3. Time main effects were not significant after FDR correction for all primary outcomes, which indicates no change over time among control group participants. Condition main effects were only significant for substance use (*b* = − 0.57, *p* = 0.002), meaning that at baseline, after controlling for age, participants in the intervention condition were less likely to utilise substance use as a coping strategy than the control condition.

**Table 2:** Descriptive statistics (N = 138)

Variable	Intervention (n = 97)				Control (n = 41)			
	Pre-test		Post-test		Pre-test		Post-test	
	*M*	*SD*	*M*	*SD*	*M*	*SD*	*M*	*SD*
Depression	39.92	12.07	35.76	12.20	39.30	9.92	40.93	11.49
SA—challenge	3.03	1.02	3.84	0.90	3.20	0.89	3.33	0.80
SA—threat	4.07	0.64	3.68	0.74	4.18	0.60	4.22	0.66
SA—resources	3.64	0.86	4.11	0.87	3.49	1.07	3.59	0.96
Cope—active coping	2.76	0.75	2.88	0.74	2.85	0.74	2.79	0.55
Cope—substance use	1.60	0.89	1.55	0.78	2.25	1.15	2.15	1.06
Cope—emotional support	2.51	0.75	2.98	0.81	2.90	0.95	2.79	0.92
Cope—instrumental support	2.55	0.74	2.89	0.81	2.98	0.91	2.67	0.81
Cope—behavioural disengagement	2.38	0.86	2.15	0.82	2.44	0.70	2.36	0.69
Cope—positive framing	2.29	0.86	2.69	0.76	2.78	0.85	2.60	0.89
Cope—planning	2.76	0.86	3.07	0.78	2.98	0.64	2.78	0.71
Cope—humour	2.58	1.04	2.82	1.00	2.89	1.05	2.72	0.94
Cope—self-blame	3.29	0.85	2.99	0.89	3.54	0.69	3.44	0.70
Reflective coping	2.75	0.67	2.95	0.66	2.85	0.48	2.78	0.56
Hope—agency	4.42	1.79	5.25	1.68	4.74	1.49	4.72	1.58
Hope—pathway	5.13	1.57	5.79	1.42	5.44	1.42	5.27	1.27

**Table 3:** Linear multilevel model results (N = 138)

Variable	Fixed effects				Control (n = 41)		Intervention (n = 97)	
	Time	Condition	Time* Condition (95% CI)	Effect size (d)	Intercept	Slope (95% CI)	Intercept	Slope (95% CI)
Depression	1.63	− 0.69	− 5.79** (− 9.29, − 2.28)	0.56	40.22	1.63 (− 1.31, 4.56)	39.53	− 4.16*** (− 6.07, − 2.25)
SA—challenge	0.13	− 0.78	0.67*** (0.34, 1.01)	0.68	3.14	0.13 (− 0.15, 0.41)	3.06	0.80*** (0.62, 0.99)
SA—threat	0.03	− 0.14	− 0.43** (− 0.69, − 0.17)	0.56	4.20	0.03 (− 0.18, 0.25)	4.06	− 0.39*** (− 0.53, − 0.25)
SA—resources	0.09	0.15	0.38* (0.07, 0.69)	0.42	3.49	0.09 (− 0.17, 0.35)	3.64	0.47*** (0.31, 0.64)
Cope—active coping	− 0.06	− 0.01	0.18 (− 0.08, 0.45)	0.23	2.79	− 0.07 (− 0.29, 0.16)	2.78	0.12 (− 0.03, 0.26)
Cope—substance use	− 0.04	− 0.57**	− 0.02 (− 0.31, 0.27)	0.02	2.19	− 0.04 (− 0.28, 0.20)	1.62	− 0.06 (− 0.21, 0.10)
Cope—emotional support	− 0.11	− 0.38*^†^	0.59*** (0.28, 0.90)	0.65	2.89	− 0.11 (− 0.37, 0.16)	2.51	0.48*** (0.32, 0.65)
Cope—instrumental support	− 0.32*^†^	− 0.39*^†^	0.67*** (0.35, 1.00)	0.71	2.95	− 0.32*^†^ (− 0.59, − 0.04)	2.56	0.35*** (0.18, 0.53)
Cope—behavioural disengagement	− 0.07	− 0.16	− 0.16 (− 0.49, 0.17)	0.17	2.51	− 0.07 (− 0.34, 0.21)	2.35	− 0.23* (− 0.41, − 0.05)
Cope—positive framing	− 0.18	− 0.44**^†^	0.59*** (0.30, 0.87)	0.70	2.74	− 0.18 (− 0.42, 0.06)	2.31	0.41*** (0.25, 0.56)
Cope—planning	− 0.18	− 0.11	0.49*** (0.23, 0.76)	0.64	2.89	− 0.18 (− 0.40, 0.04)	2.78	0.31*** (0.17, 0.46)
Cope—humour	− 0.13	− 0.41*^†^	0.36* (0.07, 0.64)	0.43	2.96	− 0.13 (− 0.37, 0.11)	2.55	0.23** (0.07, 0.38)
Cope—self-blame	− 0.10	− 0.27	− 0.20 (− 0.48, 0.08)	0.24	3.55	− 0.10 (− 0.34, 0.13)	3.28	− 0.30*** (− 0.45, − 0.15)
Reflective coping	− 0.07	− 0.04	0.27** (0.07, 0.47)	0.46	2.81	− 0.07 (− 0.23, 0.10)	2.77	0.20*** (0.09, 0.31)
Hope—agency	− 0.04	− 0.10	0.84** (0.37, 1.31)	0.61	4.58	− 0.04 (− 0.43, 0.36)	4.48	0.80*** (0.55, 1.06)
Hope—pathway	− 0.15	− 0.16	0.79** (0.33, 1.25)	0.59	5.33	− 0.15 (− 0.53, 0.24)	5.17	0.64*** (0.40, 0.89)

Age (centred by mean of the whole sample) was included in the model as a covariate variable. Cohen’s *d*s were estimated based on the t statistics of interaction terms (.2 = small; .5 = medium; .8 = large). All intercepts were significant at p < .001. *p < .05; **p < .01; ***p < .001. ^†^Not significant after false discovery rate correction

The interactions between time and condition were significant (after FDR correction) for twelve outcome measures, suggesting that the intervention had differential impacts on the primary outcomes compared to the waitlisted control over time. The estimations of the interaction terms for the twelve variables were: depression (b = − 5.79, p = 0.001), stress appraisal—challenge (b = 0.67, p < 0.001), stress appraisal—threat (b = − 0.43, p = 0.001), stress appraisal—resources (b = 0.38, p = 0.016), emotional support (b = 0.59, p < 0.001), instrumental support (b = 0.67, p < 0.001), positive framing (b = 0.59, p < 0.001), planning (b = 0.49, p < 0.001), humour (b = 0.36, p = 0.014), reflective coping (b = 0.27, p = 0.009), hope—agency (b = 0.84, p = 0.001) and hope—pathway (b = 0.79, p = 0.001). For all outcomes with statistically significant interaction terms, the Cohen’s *d*s ranged from 0.42 to 0.70, indicating medium to large effect sizes (Table 3).

Given the significance of the interaction terms, the slopes (rates of change over time) were estimated to determine the differences. Compared to waitlist control participants who showed no significant changes on all outcomes, intervention participants demonstrated significant improvements at post-test. Specifically, AFFIRM participants had significant reduction in depression (b = − 4.16, p < 0.001) and were less likely to appraise stress as threat (b = − 0.39, p < 0.001) after the intervention. They also reported increases in appraising stress as challenge (b = 0.80, p < 0.001), having the resources to deal with stress (b = 0.47, p < 0.001), emotional support (b = 0.48, p < 0.001), instrumental support (b = 0.35, p < 0.001), positive framing (b = 0.41, p < 0.001), planning (b = 0.31, p < 0.001), humour (b = 0.23, p = 0.004), reflective coping (b = 0.20, p < 0.001), and hope [agency (b = 0.80, p < 0.001) and pathway (b = 0.64, p < 0.001)] after the intervention.

### Feasibility

Out of the 249 SGMY who were screened to be eligible, 59% (*n* = 147) participants agreed to participate, completed pre-intervention assessments, and were allocated to the intervention (*n* = 106) or the waitlisted control (*n* = 41), with a total of 97 (91.5%) participants completing AFFIRM. All groups were held in-person and concluded prior to lockdown measures due to the COVID-19 pandemic. Nineteen community organisations were approached to host AFFIRM, of which six (32%) have implemented AFFIRM two or more times, six (32%) have implemented it once, and seven (36%) have not yet implemented it due to space, scheduling, or other capacity concerns. All organisations approached have expressed interest to start or continue implementation.

### Acceptability

On a standardised satisfaction scale at post-intervention with all youth who completed AFFIRM (n = 97), 96.9% (*n* = 94) of participants agreed that they learned a lot from AFFIRM, 99.0% (*n* = 96) of participants agreed that AFFIRM was helpful and applicable to their life and 100% (*n* = 97) of participants agreed that AFFIRM helped them think about how feelings, actions, and thoughts are connected. Open-ended responses stated that AFFIRM helped them learn coping skills, they felt supported by the group, the facilitators were skilled, and that the experiential activities were beneficial.

## Discussion

The aim of this study was to evaluate the efficacy of AFFIRM, an affirmative cognitive behavioural group intervention for SGMY delivered in community settings. Compared to a waitlisted control, AFFIRM participants reported significantly reduced depression and threat appraisals and improved coping and hope, as well as increased challenge and resource appraisal. The findings build on the results of the open pilot feasibility study and suggest that AFFIRM is an effective intervention for SGMY. This study aligns with encouraging findings from studies of affirmative CBT with gay and bisexual men [20] and sexual minority women [76] and contributes to the research and practice literature in four key ways: (a) identifying the impact of affirmative CBT on depression and coping outcomes among SGMY; (b) testing novel resilience-focused outcomes that align with the AFFIRM curricula; (c) implementing AFFIRM across multiple and varied community sites to participants with a range of identities; and (d) using a research design that maximises potential participant enrolment, an important component given SGMY vulnerabilities.

Overall, participants in AFFIRM reported significant decreases in depressive symptoms, similar to the outcomes of affirmative CBT trials [20]. The findings underscore the likely importance of targeting participants’ appraisals of minority stressors as a strategy for enhancing coping and reducing depression. In the pilot investigation [25], as well as this study, participant reductions in threat appraisals and increases in challenge appraisals and resources following AFFIRM suggest an enhanced ability to appraise stressful situations, thereby reducing accompanying levels of psychological distress. As cognitive appraisals of stressors ultimately influence stress responses, which can impair well-being [77], these findings are particularly encouraging. This study adds to the intervention literature by exploring the impact of key AFFIRM constructs (emotional support, instrumental support, positive framing, planning, hope) that are important to mental health but have not been extensively explored with SGMY. The effects of AFFIRM were similar to other CBT interventions which found that universal processes were effective targets of change with SGMY populations [20] while delivering an intervention drawing on minority stress theory and testing multiple change processes [78]. For example, AFFIRM works with participants to simultaneously support youths’ identities while cultivating key mechanisms for change such as positive framing and planning in order to foster greater hope for the future [79]. Given the particularly troubling rates of hopelessness and suicidality among SGMY [2, 28], this study’s positive outcomes are promising.

This study extends findings regarding the applicability and efficacy of AFFIRM to include young adults as well as youth ages 14–18. Thus, the need for interventions aimed at building adaptive coping skills within the context of an identity affirming therapeutic context is not limited to adolescents but remains relevant for SGM young adults navigating minority stressors. In particular, the group-based intervention model is an innovative approach to delivering mental health care to SGMY that has potential to improve outcomes beyond individual therapy. Groups for SGMY have been found to improve social connectedness, develop community and individual support and allow for mutual aid and reflection that highlights the universality of shared experiences [78]. As a voluntary program, AFFIRM successfully engaged both sexual and gender minority youth and young adults across a wide range of mental health and community-based settings. Our positive outcomes, along with high rates of acceptability, underscore the utility of CBT-based approaches that affirm the multidimensional spectrum of SGM experiences and identities, attend to minority stressors, actively target maladaptive responses to stressors, and build skills to proactively cope [81].

Study findings underscore the feasibility of partnering with community agencies to conduct an intervention trial. Consistently high acceptability scores indicate that AFFIRM was successfully implemented across numerous community sites with a range of facilitators. AFFIRM provides a model that supports the capacity of organisations to provide an empirically based intervention to SGMY with diverse experiences, which may begin to address the evidence-to-practice gap [82]. AFFIRM’s collaborative implementation effort involved pairing a facilitator employed by the intervention’s founders and a facilitator at the collaborating site, who received equitable training and supervision. This balancing of capacity-building with the delivery of an evidence-based intervention is complex but seeks to leverage the strengths of a community organization (typically access to and knowledge of the population) with the interventionists (curriculum and mental health expertise) to benefit SGMY. Ultimately, this process may enable community sites to strengthen their ability to deliver mental health services and further identify the importance of partnering with mental health experts, even in voluntary or part-time positions. This capacity-building approach may be an implementation practice that encourages greater fidelity and sustainability than the traditional recommendation of producing a manual and encouraging its adaptation in a more passive fashion, such as through publication and presentation [83]. Compared with similar studies, albeit with different psychosocial outcomes [72], AFFIRM had slightly better rates of enrolment and completion, and experienced only mild drop-off rates (8.5%). Engagement may have been facilitated by between session contact (such as reminder texts), the action plan components, the identity affirming group context, and the tailoring of CBT to the specific experiences of SGMY. In rapidly evolving mental health and social service contexts, is important to consider how to continue to foster ongoing engagement and retention of SGMY.

Several limitations must be noted. The study design did not involve randomisation due to community concerns and capacity. Without randomisation, this trial is at risk of allocation and selection bias. Further, as participants were waitlisted based on factors such as immediate availability of the group in their rural location or changes to their schedule, they may have felt some sense of apathy about ever being offered AFFIRM which could have contributed to attrition once they were eventually offered the intervention [84]. Yet the intervention and waitlist conditions showed no significant difference in baseline scores and demographic characteristics, suggesting a matched sample. However, differences in community resources are not known. Participants were not required to be “out” to others to participate in the intervention, however, given the recruitment based on SGMY identity, it is presumed that some awareness of themselves as SGMY was necessary. Second, although the drop-off rates from screening to enrolment to allocation are lower than in other similar studies [72], some enrolled participants did not complete AFFIRM. Future studies should consider virtual adaptations of the AFFIRM group model, which may lead to better engagement and retention, as well as provide access for SGMY prevented from accessing in-person services. Third, with only baseline versus post-test comparisons, we were not sure whether the positive effects of the intervention would persist; long-term follow-up would provide further evidence to the intervention’s efficacy. Fourth, the waitlist design does not allow for comparison with another treatment (e.g., non-adapted CBT). Fifth, AFFIRM draws on minority stress theory but this study does not specifically test those constructs. Although Pachankis et al. [20] identified encouraging, yet non-significant trends in minority stressors (e.g., rejection sensitivity, concealment) in their affirmative CBT study, future studies of AFFIRM should explicitly include relevant measures. Finally, this study did not assess feasibility and acceptability from the community organisations' perspective.

This study responds to the call for tailored, empirically based interventions to address the striking mental health disparities of SGMY [36]. As a brief CBT intervention, AFFIRM was simultaneously able to attend to the universal and minority stressors that threaten the mental health of SGMY while targeting several resilience enhancing processes. These results illustrate the role of AFFIRM in disrupting the psychosocial stress trajectory of SGMY by affirming youths’ SGM identities and fostering the development of adaptive coping skills.

## Supplementary Information

**Additional file 1.** AFFIRM Core Survey Items.

## Data Availability

Data may be available, pending consultation with the University of Toronto’s Health Sciences Research Ethics Board (REB). Data requests may be sent to the principal investigator at shelley.craig@utoronto.ca, who will consult with the REB.

## Abbreviations

CBT: Cognitive behavioural therapy
SGMY: Sexual and gender minority adolescents and young adults
