# Supplementary material for: Efficacy of affirmative cognitive behavioural group therapy for sexual and gender minority adolescents and young adults in community settings in Ontario, Canada
Source: BMC Psychol. 2021 Jun 7;9:94. doi: 10.1186/s40359-021-00595-6 (PMC8183324; doi:10.1186/s40359-021-00595-6)
Supplement: Supplementary file 1 — Additional file 1. AFFIRM Core Survey Items. [file 40359_2021_595_MOESM1_ESM.docx]

**Efficacy of Affirmative Cognitive Behavioural Group Therapy for Sexual and Gender Minority Adolescents and Young Adults in Community Settings in Ontario, Canada**

Shelley L. Craig^1*^, Andrew D. Eaton^1^, Vivian W. Y. Leung^1^, Gio Iacono^2^, Nelson Pang^1^, Frank Dillon^3^, Ashley Austin^4^, Rachael Pascoe^1^, Cheryl Dobinson^5^

^1^Factor-Inwentash Faculty of Social Work, University of Toronto; Toronto, ON, Canada

^2^School of Social Work, University of Connecticut; Hartford, CT, USA

^3^College of Integrative Sciences and Arts, Arizona State University; Tempe, AZ, USA

^4^Ellen Whiteside-McDonnell School of Social Work, Barry University; Miami, FL, USA

^5^Planned Parenthood Toronto; Toronto, ON

*Corresponding author. Factor-Inwentash Faculty of Social Work, University of Toronto. 246 Bloor St. W., Toronto, ON, Canada, M5S1V4 Phone: +1-416-978-8847. Email: [shelley.craig@utoronto.ca](mailto:shelley.craig@utoronto.ca)

**Supplementary File 1 – AFFIRM Participant Survey**

Stress Appraisal Measure for Adolescents (SAMA)

What about stress in your life? The following questions are about the stress you experience in your daily life. This will help us understand what you might be dealing with. In my DAILY LIFE...

|  | Strongly agree | Somewhat agree | Neither agree nor disagree | Somewhat disagree | Strongly disagree |
| --- | --- | --- | --- | --- | --- |
| I have the ability to overcome stress. |  |  |  |  |  |
| I perceive stress as threatening. |  |  |  |  |  |
| There is someone I can turn to for help. |  |  |  |  |  |
| I know how to decrease my stress in a positive way. |  |  |  |  |  |
| I have what it takes to beat stress. |  |  |  |  |  |
| I feel anxious. |  |  |  |  |  |
| Stressful events impact me greatly. |  |  |  |  |  |
| There is help available to me. |  |  |  |  |  |
| The outcome of stressful events is negative. |  |  |  |  |  |
| Stressful events have serious implications for my life. |  |  |  |  |  |
| I have the resources available to me to overcome stress. |  |  |  |  |  |
| Stress has a negative impact on me. |  |  |  |  |  |
| There are long-term consequences as the result of stress. |  |  |  |  |  |

Brief COPE

When stressed, generally, in my DAILY LIFE...

|  | Not at all | A little bit | Some | A lot |
| --- | --- | --- | --- | --- |
| I turn to work or other activities to take my mind off things. |  |  |  |  |
| I concentrate my efforts on doing something about the situation I'm in. |  |  |  |  |
| I say to myself "this isn't real". |  |  |  |  |
| I use alcohol or other drugs to make myself feel better. |  |  |  |  |
| I get emotional support from others. |  |  |  |  |
| I give up trying to deal with it. |  |  |  |  |
| I take action to try to make the situation better. |  |  |  |  |
| I refuse to believe that it has happened. |  |  |  |  |
| I say things to let my unpleasant feelings escape. |  |  |  |  |
| I get help and advice from other people. |  |  |  |  |
| I use alcohol or other drugs to help me get through it. |  |  |  |  |
| I try to see it in a different light, to make it seem more positive. |  |  |  |  |
| I criticize myself. |  |  |  |  |
| I try to come up with a strategy about what to do. |  |  |  |  |
| I get comfort and understanding from someone. |  |  |  |  |
| I give up the attempt to cope. |  |  |  |  |
| I look for something good in what is happening. |  |  |  |  |
| I make jokes about it. |  |  |  |  |
| I do something to think about it less, such as going to the movies, watching TV, reading, daydreaming, sleeping, or shopping. |  |  |  |  |
| I accept the reality of the fact that it happened. |  |  |  |  |
| I express my negative feelings. |  |  |  |  |
| I try to find comfort in my spirituality. |  |  |  |  |
| I try to get advice or help from other people about what to do. |  |  |  |  |
| I learn to live with it. |  |  |  |  |
| I think hard about what steps to take. |  |  |  |  |
| I blame myself for things that happened. |  |  |  |  |
| I reflect, mediate or pray. |  |  |  |  |
| I make fun of the situation. |  |  |  |  |

Proactive Coping – Reflective Coping Subscale

The following statements deal with reactions you may have to various situations. Indicate how true each of these statements are depending on how you feel about the situation. This helps us understand how you deal with problems. When stressed, generally, in my DAILY LIFE...

|  | Not at all true | Barely true | Somewhat true | Completely true |
| --- | --- | --- | --- | --- |
| I take action only after thinking carefully about a problem. |  |  |  |  |
| I make lists and try to focus on the most important things first. |  |  |  |  |
| In my mind I go through many different scenarios in order to prepare myself for different outcomes. |  |  |  |  |
| I think about every possible outcome to a problem before tackling it. |  |  |  |  |
| I imagine myself solving a difficult problem before I actually have to face it. |  |  |  |  |
| When I have a problem with my teachers, friends, or family I imagine beforehand how I will deal with them successfully. |  |  |  |  |
| When there are serious misunderstandings with teachers, family members or friends, I practice before how I will deal with them. |  |  |  |  |
| Rather than acting impulsively, I usually think of various ways to solve a problem. |  |  |  |  |
| I imagine myself solving difficult problems. |  |  |  |  |
| I address a problem from various angles until I find the appropriate action. |  |  |  |  |
| Before tackling a difficult task, I imagine being successful in doing it. |  |  |  |  |

Beck’s Depression Inventory (BDI-III)

What about your feelings?

In the past **TWO (2) WEEKS**,

- I do not feel sad
- I feel sad
- I am sad all the time and I can't snap out of it
- I am so sad and unhappy that I can't stand it

In the past **TWO (2) WEEKS,**

- I am not particularly discouraged about the future
- I feel discouraged about the future
- I feel I have nothing to look forward to
- I feel the future is hopeless and that things cannot improve

In the past **TWO (2) WEEKS**,

- I do not feel like a failure
- I feel I have failed more than the average person
- As I look back on my life, all I can see is a lot of failures
- I feel I am a complete failure as a person

In the past **TWO (2) WEEKS**,

- I get as much satisfaction out of things as I used to
- I don't enjoy things the way I used to
- I don't get real satisfaction out of anything anymore
- I am dissatisfied or bored with everything

In the past **TWO (2) WEEKS**,

- I don't feel particularly guilty
- I feel guilty a good part of the time
- I feel quite guilty most of the time
- I feel guilty I am being punished

In the past **TWO (2) WEEKS**,

- I don't feel disappointed in myself
- I am disappointed in myself
- I am disgusted with myself
- I hate myself

In the past **TWO (2) WEEKS,**

- I don't feel I am any worse than anyone else
- I am critical of myself for my weaknesses or mistakes
- I blame myself all the time for my faults
- I blame myself for everything bad that happens

In the past **TWO (2) WEEKS,**

- I don't have any thoughts of killing myself
- I have thoughts of killing myself, but I would not carry them out
- I would like to kill myself
- I would kill myself if I had the chance

In the past **TWO (2) WEEKS,**

- I don't cry any more than usual
- I cry more now than I used to
- I cry all the time now
- I used to be able to cry, but now I can't cry even though I want to

In the past **TWO (2) WEEKS,**

- I am no more irritated by things than I ever was
- I am slightly more irritated now than usual
- I am quite annoyed or irritated in other people
- I feel irritated all the time

In the past **TWO (2) WEEKS,**

- I have not lost interest in other people
- I am less interested in other people than I used to be
- I have lost most of my interest in other people
- I have lost all of my interest in other people

In the past **TWO (2) WEEKS,**

- I make decisions about we well as I ever could
- I put off making decisions more than I used to
- I have greater difficulty in making decisions more than I used to
- I can't make decisions at all anymore

In the past **TWO (2) WEEKS,**

- I don't feel that I look any worse than I used to
- I am worried that I am looking old or unattractive
- I feel there are permanent changes in my appearance that make me look unattractive
- I believe that I look ugly

In the past **TWO (2) WEEKS,**

- I can work about as well as before
- It takes an extra effort to get started at doing something
- I have to push myself very hard to do anything
- I can't do any work at all

In the past **TWO (2) WEEKS,**

- I can sleep as well as usual
- I don't sleep as well as I used to
- I wake up 1-2 hours earlier than usual and find it hard to get back to sleep
- I wake up several hours earlier than I used to and cannot get back to sleep

In the past **TWO (2) WEEKS,**

- I don't get more tired than usual
- I get tired more easily than I used to
- I get tired from doing almost anything
- I am too tired to do anything

In the past **TWO (2) WEEKS,**

- My appetite is no worse than usual
- My appetite is not as good as it used to be
- My appetite is much worse now
- I have no appetite at all anymore

In the past **TWO (2) WEEKS,**

- I haven't lost much weight, if any, lately
- I have lost more than five pounds
- I have lost more than ten pounds
- I have lost more than fifteen pounds

In the past **TWO (2) WEEKS,**

- I am not more worried about my health than usual
- I am worried about physical problems and it's hard to think of much else
- I am very worried about physical problems and it's hard to think of much else
- I am so worried about my physical problems that I cannot think of anything else

In the past **TWO (2) WEEKS,**

- I have not noticed any recent change in my interest in sex
- I am less interested in sex than I used to be
- I have almost no interest in sex anymore
- I have lost interest in sex completely

Hope Scale

What about the future?

The following questions give us some insight about how you feel about your present and future.

|  | Definitely false | Mostly false | Somewhat false | Slightly false | Slightly true | Somewhat true | Mostly true | Definitely true |
| --- | --- | --- | --- | --- | --- | --- | --- | --- |
| I think of many ways to get out of a jam. |  |  |  |  |  |  |  |  |
| I energetically pursue my goals. |  |  |  |  |  |  |  |  |
| I feel tired most of the time. |  |  |  |  |  |  |  |  |
| There are lots of ways around any problem. |  |  |  |  |  |  |  |  |
| I am easily downed in an argument. |  |  |  |  |  |  |  |  |
| I can think of many ways to get the things in life that are important to me. |  |  |  |  |  |  |  |  |
| I worry about my health. |  |  |  |  |  |  |  |  |
| Even when others get discouraged, I know I can find a way to solve the problem. |  |  |  |  |  |  |  |  |
| My past experiences have prepared me well for my future. |  |  |  |  |  |  |  |  |
| I've been pretty successful in life. |  |  |  |  |  |  |  |  |
| I usually find myself worrying about something. |  |  |  |  |  |  |  |  |
| I meet the goals that I set for myself. |  |  |  |  |  |  |  |  |
